# Adolescent condom use in Southern Africa: narrative systematic review and conceptual model of multilevel barriers and facilitators

**DOI:** 10.1186/s12889-021-11306-6

**Published:** 2021-06-26

**Authors:** Áine Aventin, Sarah Gordon, Christina Laurenzi, Stephan Rabie, Mark Tomlinson, Maria Lohan, Jackie Stewart, Allen Thurston, Lynne Lohfeld, G. J. Melendez-Torres, Moroesi Makhetha, Yeukai Chideya, Sarah Skeen

**Affiliations:** 1 School of Nursing and Midwifery, Queen’s University Belfast, Belfast, Northern Ireland, UK; 2 Institute for Life Course Health Research, Stellenbosch University, Stellenbosch, South Africa; 3 Department of Psychiatry and Mental Health, HIV Mental Health Research Unit, Cape Town, South Africa; 4 School of Education, Social Sciences, Education and Social Work, Queen’s University Belfast, Belfast, Northern Ireland; 5 Centre for Public Health, Queen’s University Belfast, Belfast, Northern Ireland; 6 College of Medicine and Health, University of Exeter, Exeter, England, UK; 7 World Vision, Maseru, Lesotho

**Keywords:** Systematic review, Condom use, Sexual and reproductive health, Adolescents, Adolescent health, Southern Africa

## Abstract

**Background:**

Adolescent HIV and pregnancy rates in Southern Africa are amongst the highest in the world. Despite decades of sexual and reproductive health (SRH) programming targeting adolescents, recent trends suggest there is a continued need for interventions targeting condom use for this age group.

**Methods:**

This review synthesises evidence from qualitative studies that describe the determinants of condom use among adolescents in Southern Africa. We conducted systematic searches in four databases. Data were extracted, appraised for quality and analysed using a ‘best-fit’ framework synthesis approach.

**Results:**

We coded deductively findings from 23 original studies using an a priori framework and subsequently conducted thematic analysis. Synthesised findings produced six key themes relating to: 1) pervasive *unequal gender norms* and restrictive masculinities favouring male sexual decision-making and stigmatising condom use in committed relationships; 2) *other social norms* reflecting negative constructions of adolescent sexuality and non-traditional family planning; 3) *economic and political barriers* including poverty and a lack of policy support for condom use; 4) *service-level barriers* including a lack of youth-friendly SRH services and comprehensive sex education in schools; 5) *interpersonal barriers and facilitators* including unequal power dynamics in sexual partnerships, peer influences and encouraging condoning condom use, and inadequate communication about SRH from parents/caregivers; and 6) *negative attitudes and beliefs* about condoms and condom use among adolescents. A conceptual model was generated to describe determinants of condom use, illustrating individual-, interpersonal- and structural-level barriers and facilitating factors.

**Conclusion:**

SRH programming targeting barriers and facilitators of condom use at multiple levels is recommended in Southern Africa. We present a multilevel integrated model of barriers and facilitators to guide adolescent SRH decision-making, programme planning and evaluation. Given the existence of multilevel barriers and facilitators, interventions should, likewise, take a multilevel approach that incorporates locally relevant understanding of the individual-, interpersonal- and structural-level barriers and facilitators to condom use among adolescents in the region.

**Supplementary Information:**

The online version contains supplementary material available at 10.1186/s12889-021-11306-6.

## Background

In sub-Saharan Africa, consistent use of condoms among adolescents is low [1]. This is likely a contributory factor for the high prevalence of sexually transmitted infections (STIs), including HIV, and adolescent pregnancy in the region [1–4], which has persisted despite more than two decades of research and programming. As many as 20% of women in sub-Saharan Africa have their first child by the age of 19 [5], and around half of these pregnancies are unintended [6]. In 2017, adolescent girls accounted for 25% of all new global HIV infections, despite comprising only 10% of the population [7]. Further, in the Southern African region in particular, there are stark gender disparities in relation to HIV risk: risk among adolescent girls aged 15–19 is six times higher than among their male counterparts [8]. Further, a recent systematic review has identified persistent perceived and experienced barriers to STI services for adolescents [9].

Much research has attempted to uncover determinants of condom use among adolescents that might be targeted by sexual and reproductive health (SRH) programmes. A complex array of individual-, interpersonal- and structural-level influences are at play [10–14], including lack of desire to use condoms, lack of local access to condoms, gender inequalities and social norms restricting condom use, and age-disparate and transactional sexual relationships [2, 15–18]. These factors, taken together, may help explain why SRH programmes that focus only on individual determinants of condom use and ignore wider interpersonal and structural determinants have had limited impact [19]. One recent meta-analysis [20] found individual-level social-cognitive determinants accounted for only 15–30% of the variance in adolescent condom use intentions and behaviours. These findings indicate that interpersonal and social dynamics, plus context-dependent cultural and structural conditions, are key factors associated with condom use that must be explored further both theoretically and empirically [20]. In order to craft more effective condom use interventions for adolescents, programme developers must adopt a multilevel approach, including a combination of structural and behavioural interventions that target both males and females [7, 13]. Systematic reviews can aid this process by examining multilevel barriers and facilitators of condom use in particular geographic regions. Results of such work would be better positioned to guide future intervention planning and empirical work on this important subject. Qualitative studies from Southern Africa are particularly important for deriving deeper and more nuanced understandings of the barriers to condom use, and can assist researchers and practitioners develop, adapt, or scale-up interventions in the region [13, 21].

This review aims to describe the individual-, interpersonal- and structural-level determinants of condom use among male and female adolescents in Southern Africa and then develop an integrated conceptual framework. The findings provide clear recommendations for future research aiming to test causal pathways explaining condom use among adolescents, and for SRH programme development and adaptation in the Southern African region.

## Methods

The synthesis reported here is part of a larger research study to adapt an SRH intervention for adolescents in Southern Africa [22]. The project includes two linked reviews that used a common preregistered protocol [23] but reviewed qualitative and quantitative literature separately. In this paper we include qualitative studies only, in order to promote a deep exploration of salient individual and interpersonal barriers and facilitators of condom use among adolescents, as well as broader social, cultural and structural dynamics. Findings from a review of quantitative studies are reported elsewhere [24].

For the present review, the ENTREQ (Enhancing Transparency in Reporting the Synthesis of Qualitative Research) [25] guidelines informed reporting of the analysis. This review aimed to answer the following research questions: 1) *What are the individual-, interpersonal- and structural-level barriers and facilitators of condom use among adolescents in Southern Africa? 2) How can these multilevel barriers and facilitators be conceptualised within an integrated framework?*

### Guiding frameworks

‘Best-fit’ framework synthesis [26, 27] was used to guide the data analysis. The approach provides a method for incorporating relevant theories within a framework analysis and is particularly suited to the development of new conceptual models relating to health behaviours. The approach involves identifying an a priori theoretical framework based on one or more published theories or models against which review data are coded.

The current review is part of a study that involves the adaptation of an SRH intervention named *If I Were Jack* (JACK) for the Southern African context [28–30]. JACK was developed in consultation with stakeholders for use in the UK and Ireland. Its theory of change is underpinned by social cognitive theories of behaviour change plus gender-transformative theories and understandings of broader socio-cultural influences and underlying values (especially relating to religiosity and social class) [28–30]. Given the focus of the broader study, we chose to use the intervention theory of change [28] to inform the ‘best-fit’ framework*.*

Given that a further aim of the study was to examine the relevance of the *If I Were Jack* Theory of Change to the Southern African context (i.e., to examine if it was appropriate given the likelihood of contextual differences between the UK and Southern Africa), we also included in the a priori framework theoretical models that conceptualise a broad range of environmental and individual-level influences on behaviour. These included the established Social Ecological Model [31, 32], which acknowledges that individual decision-making is shaped by a dynamic interplay between features of the environment at intrapersonal, interpersonal, organisational, community and society level, as well as an integrated social-cognitive model of condom use among adolescents in sub-Saharan Africa [20], which highlights individual level factors that relate to past condom use behaviours, including attitudes about condoms and condom use, and perceptions of norms, risks, behavioural control and barriers to condom use. Together, these three models were used to generate a number of a priori themes against which the extracted data were coded (see Table 1).

**Table 1:** A priori themes reflecting theoretical determinants of condom use among adolescents from three ‘best-fit’ models

Social Ecological Model McLeroy et al. [31]; Sallis et al., [32]	Integrated model of condom use in sub-Saharan African youth Protogerou et al. [20]	***If I Were Jack*** theory of change model Aventin et al. [28]; Lohan et al. [29]	Themes for Coding Framework
**INDIVIDUAL LEVEL DETERMINANTS**			
*Intrapersonal* Knowledge, attitudes, beliefs, skills and behaviours		Knowledge about SRH, where to obtain condoms	Sexual and Reproductive Health Knowledge
	Attitudes (beliefs about the behaviour)	Attitudes (beliefs about the behaviour and positive planning)	Attitudes and beliefs about condom use
	Barriers perceived e.g. stigma, lack of pleasure, lack of effectiveness, religious beliefs and actual e.g. access to condoms and partner refusal to use condoms	Barriers (perceived) e.g. myths, stigma, pleasure, effectiveness, religious beliefs, lack of consent, sexual coercion	Perceived barriers to condom use
	Risk perception	Beliefs about consequences of unprotected sex (episodic future thinking; anticipated regret)	Risk perception and beliefs about consequences of not using condoms
	Control (beliefs about self-efficacy and perceived behavioural control)	Beliefs about capabilities (perceived behavioural control & self-efficacy communicating to partners, peers, parents, and professionals)	Beliefs about ability to obtain and use condoms
	Intentions	Intentions to avoid unprotected sex	Intentions to use condoms
	Past condom use behaviour		Past condom use behaviour
		Age, gender, socioeconomic status, religiosity	Sociodemographic influences
**INTERPERSONAL LEVEL DETERMINANTS**			
*Interpersonal* Social network and Relationships	Barriers (actual) e.g. partner refusal to use condoms	Social influences: Peers Parental values and beliefs Quality of parent-child communication	Interpersonal determinants
**STRUCTURAL LEVEL DETERMINANTS**			
*Organisational* Relevant institutions	Barriers (actual) e.g. access to condoms	Organisational influences (school, church, health services)	Organisational determinants
*Community/Society* Social norms and values	Norms	Social influences: Gender norms Social norms	Social norms and values relating to condom use
*Policy* Local and national laws and policies			Political and economic determinants

### Criteria for study inclusion

Primary or secondary qualitative studies published in English in peer-reviewed journals between January 2000 and August 2019 were included in the review. The inclusion of only English publications was due to a lack of resources for translation. We included all study designs that directly reported on relevant determinants of condom use in adolescents and were conducted in Southern African Development Community (SADC) countries (Angola, Botswana, Comoros, Democratic Republic of Congo, Eswatini, Lesotho, Madagascar, Malawi, Mauritius, Mozambique, Namibia, Seychelles, South Africa, Tanzania, Zambia and Zimbabwe). We also included studies with participants of any sex, sexual orientation and gender identity aged 13 to 19. We chose this age range as the intervention that is the focus of the broader study is aimed at teenagers aged 13–16. If a study included participants outside this age range, we sought to examine outcomes for those participants within the 13–19 age range only. If this was not possible, we included the full study if the mean age, or at least half the participants, fell within this age range. We included only studies that reported outcomes relating to condom use by adolescents. These criteria informed the development of the search terms and screening of records.

### Search strategy and screening methods

**Fig. 1:**
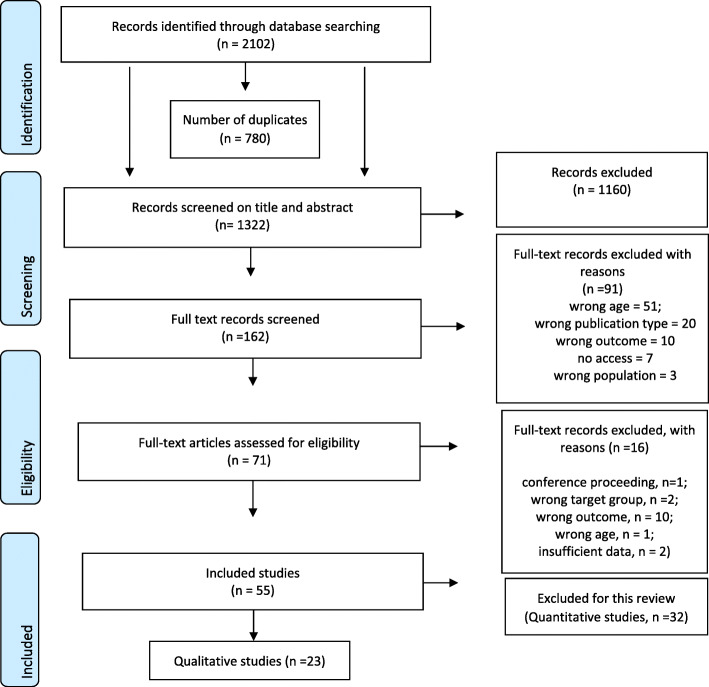
PRISMA Flowchart

We searched four databases - MEDLINE, PsycINFO, Embase as recommended by Cochrane [33] - supplemented with the Web of Science to ensure any possible bias in database search algorithms was minimised. The search terms used are detailed in Additional File 1. Duplicates were removed prior to review. As per our review protocol [23], we independently screened titles, abstracts and full text articles in duplicate, applying the study eligibility criteria. If there were multiple published reports for a study, we merged the information from these publications to provide a single record of the study’s data. We used the software programme Rayyan [34] for record management and screening. The inclusion and exclusion of records were recorded in a PRISMA flow diagram (Fig. 1) [35].

### Data extraction

We captured all necessary information from the included studies using a pre-designed data extraction form. For each eligible study, one review author (SG) extracted data, including primary data extracts, themes presented by authors and author commentary about those data and/or themes. This extraction was checked by a second author (SR or CL) for a random sample of 25% of the included studies (6 studies in total) after data from the first ten studies had been extracted Discrepancies were identified, recorded and resolved through discussion. Study characteristics entered onto the form include: study setting, inclusion and exclusion criteria, sample characteristics, research design and analysis methods, type and details of intervention, as well as narrative direct quotations or summaries of quotations relating to determinants of condom use.

Data consisted of adolescent self-reports, or participants’ perceptions of determinants of adolescent condom use. Outcomes were categorised as behavioural outcomes (i.e., condom use, access to condoms) or cognitive outcomes (i.e., outcome relating to attitudes, beliefs or knowledge about condom use). Within each sub-category, we further determined whether the outcomes assessed were individual-, interpersonal- or structural-level determinants.

### Quality appraisal

One reviewer (SG) assessed the quality of each included study using the Joanna Briggs Institute (JBI) critical appraisal checklist for qualitative studies [36], which yielded an overall classification of high or low quality for each study. Studies were classified as being of low quality if more than three domains (out of a possible 10) were missing. Assessments were checked by a second reviewer (CL). Although no studies were excluded from the review based on quality, they did widely vary, so quality classifications were included in the interpretation of findings.

### Data synthesis

‘Best-fit’ framework synthesis [26, 27] was used to guide the analysis because it provided a method for incorporating the identified theories within a framework analysis and provided a framework for development of a new conceptual model. It incorporates both deductive and inductive analysis. Qualitative data extracts were coded against the 12 a priori concepts from the ‘best-fit’ model using NVivo 11. We entered concept headings (see Additional File 2) into NVivo and data were coded deductively under the relevant headings. All data fit within the a priori framework*.* Subsequently, we revisited the evidence to explore the relationships between a priori concepts. Using thematic analysis, we clustered and synthesised the 12 a priori concepts into a final set of six themes and used them to develop a conceptual model of the barriers and facilitators of condom use among adolescents in Southern Africa. The analysis was led by one author (ÁA), with discussions held with the broader team at each stage until consensus was reached.

In the results section below, all research participant statements (quotes) appear in italics to easily distinguish them from study author views and statements, which are presented in italics without quotation marks. Also, any words removed from a quote to enhance readability are shown by ellipses (…) and added words are provided in square brackets.

## Results

### Search results

We identified 2102 records, of which 780 were duplicates. Following screening of the remaining 1322 records, 23 qualitative studies were included in the review (see PRISMA flowchart Fig. 1).

### Characteristics of the selected studies

The characteristics of the included studies are summarised in Table 2. Studies from 11 countries in Southern Africa were included. Sample size was reported in all but one of the studies and ranged from 16 to 255, and resulting in a reported total of 2270 participants. Two studies included participants from four countries outside of Southern Africa, however, only data from included countries was extracted. Age range was reported in 22/23 studies and ranged from 10 to 24 years old, with mean age within review parameters. Reporting of participant sex/gender in the included studies was sporadic (*n* = 12; 57.1%). Eight studies were single sex (*n* = 5 included girls only and *n* = 3 included boys only). The remaining four studies that reported sex of participants included roughly an equal number of females and males.

**Table 2:** Included study characteristics

Author and year	Study design	Study aim	Country	Study population description	Female (n, %)	Male (n, %)	Total sample (n) at start of study	Age range
Baumgartner et al., 2010 [37]	Qualitative	To understand how Tanzanian adolescents think about and understand the concepts of faithfulness and partner reduction in the context of both HIV and pregnancy prevention	Tanzania	This study included 20 focus group discussions (FGDs) with 158 adolescents, aged 14 20			158	14–20
Bosmans et al., 2006 [38]	Qualitative	To explore adolescents’ access to condom information and supplies.	Democratic Republic of Congo	Focus group discussions were conducted with 11 groups of adolescents. Two interviews were conducted with programme officers of one SRH peer education programme. In addition they had an focus group with a group of 34 adolescent peer educators in Bukavu.	60%	40%	117	
Butts et al., 2018 [39]	Qualitative	To identify sources of HIV prevention knowledge among young women aged 10–14 years and community-based strategies to enhance HIV prevention in Zambia.	Zambia	Focus group discussions were conducted with 114 young women in Zambian provinces with the highest rates (~ 20%) of HIV	100%		114	10–14
Capurchande, et al., 2016 [40]	Qualitative	To explore adolescents and young adults’ experiences with contraception in Mozambique	Mozambique	Four focus group discussions, 16 in-depth interviews, four informal conversations, and observations were equally divided between two study sites.			62	15–24
Casale,et al., 2010 [41]	Qualitative	To explore the complexities facing a faith based organization during its delivery of an HIV-prevention programme for adolescents.	South Africa	11 focus group discussions: two groups with parents (*n* = 34), two with teachers (*n* = 16), four with learners (*n* = 48) and three with programme facilitators (*n* = 6).			104	
Cockcroft et al., 2010 [42]	Qualitative	To explore community views of inter-generational sex	Botswana, Namibia and Swaziland	The study included 12 focus group discussions with women aged 15–24 years			between 60 and 120	15–24
Groes-Green et al., [43]	Qualitative	To examine how class, gender and peer education affects safe sex in male youth and identifies the reasons behind condom use	Mozambique	52 boys who qualified as consistent condom users between the ages of 18 and 23			52	18–23
Klinger & Ramin, 2017 [44]	Qualitative	Evaluate perceptions, attitudes, and misconceptions regarding STIs and contraception among female and male adolescents	Madagascar	Data was collected from female and male adolescents ages 15–19 years (*n* = 43) in Northern Madagascar in 2014 using focus group discussions	53%	47%	43	15–19
Lusey,et al., 2014 [45]	Qualitative	To explore discourses of young churchgoers from deprived areas of Kinshasa regarding masculinity and sexuality in the era of HIV.	Congo	This study included 16 semi-structured interviews with unmarried young churchgoers		16	16	15–24 (mean 19.6)
MacPhail & Campbell, 2001 [46]	Qualitative	To increase our understandings of the influences on adolescent sexuality within a broader interest in HIV-prevention in Southern Africa.	South Africa	Study informants comprised 44 young women and men in the 13–25 year age group.	50%	50%	44	13–25
Manuel, 2005 [47]	Qualitative	To explore how urban youth in Mozambique perceive their sexual behaviour and identifies the factors that hinder them from having safer sex in the context of HIV/AIDS, with special emphasis on the condom use.	Mozambique	Data was collected from high school students in Maputo, Mozambique. Using a combination of focus group discussions, interviews and informal conversations			Not reported	16–18
Mavhu et al., [48]	Qualitative	Follow on to a quantitative survey that sought to characterize male sexual partners and sexual behaviours of sexually active HIV positive AGYW in Zimbabwe.	Zimbabwe	In-depth interviews were conducted with purposively sampled 28 adolescent girls and young women (16–24 years).	100%		28	16–24
McCleary-Sills, et al., 2013 [49]	Qualitative	To examine Gendered norms, sexual exploitation and adolescent pregnancy in rural Tanzania	Tanzania	A participatory research and action project (Vitu Newala) conducted formative research in a rural district on the dynamics of sexual risk and agency among 82 girls aged 12–17.	100%		82	12–17
McHome et al., 2015 [50]	Qualitative scripted scenarios	To examine staff perceptions of adolescent sexual health and reproductive services in Tanzania	Tanzania	Health service staff from 33 health care facilities			Between 41 and 48	18–19
Meekers et al., 2001 [51]	Qualitative	To understand constraints to adolescent condom procurement. Including condom use negotiation, consistency of condom use, and condom distribution amongst adolescents.	Botswana	Eight focus groups were conducted which included four to six participants per group (male and female) between the ages of 14–20.			Between 32 and 48	14–20
Moyo & Rusinga, 2017 [52]	Qualitative	To understand the importance of reproductive health education to contraceptive use among adolescents	Zimbabwe	A total of 185 adolescents aged 15–19 years were sampled.	96	89	185	15–19 (mean 17)
Mulumeoderhwa, 2018 [53]	Qualitative	To investigate young men’s perspectives about condom use, concurrent sexual partnerships and sex in the context of HIV/AIDS.	Democratic Republic of Congo	28 boys aged 16–20 from two urban and two urban high schools in South Kivu provinces.		100%	28	16–20
Mwalabu et al., 2017 [54]	Qualitative	To explore the sex and relationship experiences of young women growing up with perinatally-acquired HIV in order to understand how to improve SRH care and associated outcomes	Malawi	Data was collected for 14 cases through in-depth interviews (i.e. a total of 42 participants)	100%		42	15–19
Nash et al., 2019 [55]	Qualitative	To understand how and what sexual and reproductive health information is shared with girls, in Malawi, and perceptions of such information among key informants	Malawi	Forty semi-structured interviews were conducted with three participant groups: adolescent girls (*n* = 18), mothers/female guardians of adolescent girls (n = 12), and leaders of initiation rites (*n* = 10).			40	10–18
Self et al., 2018 [56]	Qualitative	To explore the perspectives of youth and adults about the drivers and barriers to youth accessing family planning in Malawi and their ideas to improve services	Malawi	34 focus groups were conducted with youth 34 FGDs with 255 youth and 40 parent/guardian participants.			255	15–24
Sommer et al., 2015 [57]	Qualitative	To explored the masculinity norms shaping pubescent boys perceptions of and engagement in (unsafe) sexual behaviours	Tanzania	In-depth interviews with adolescent boys in and out of school, key informant interviews (e.g. parents, teachers, religious leaders), focus groups with teachers, and participatory activities with adolescent boys in and out of school (*n* = 160)		100%	160	16–19
Sommer et al., 2019 [58]	Qualitative	To explore structural and environmental factors influencing young people’s access to and use of alcohol, and subsequent engagement in safe or unsafe sexual behaviours, from their own perspectives	Tanzania	The study included 177 adolescent girls and boys in and out of school in four sites across Dar es Salaam, Tanzania.			177	15–19
Winskell, et al., 2011 [59]	Qualitative	In order to inform education and communication efforts to increase condom use, we examined social representations of condoms among young people aged 10–24 in six African countries/regions with diverse HIV prevalence rates	Swaziland, Namibia, Kenya, South-East Nigeria, Burkina Faso, and Senegal	A unique data source was used, namely 11,354 creative ideas contributed from these countries to a continent-wide scriptwriting contest, held from 1st February to 15th April 2005, on the theme of HIV/AIDS. We stratified each country sample by the sex, age (10–14, 15–19, 20–24), and urban/rural location of the author and randomly selected up to 10 narratives for each of the 12 resulting strata, netting a total sample of 586 texts for the six countries.			Not clear (586 texts)	10–24

### Quality of included studies

Overall, the quality of studies was found to be high, with notable exceptions.

Five of the 10 domains were scored “Yes” (present) across all 23 studies and covered issues related to statements about research methodology and question; correct methodology employed; representation of data and analysis; interpretation of results; and representation of participant voices. Only one study (Bosmans et al., 2006) received a “No” (not present) for conclusions flowing from data interpretation and analysis. Two domains were slightly more mixed: 6/23 studies (26%) did not have congruency between the stated philosophical perspective and the research methodology and another six did not report ethical research criteria and evidence of ethical approval by an appropriate body.

In contrast with these overall positive findings, two of the domains in the assessment tool, related to theoretical and/or cultural positionality of the researcher, and accounting for the influence of the researcher on research itself, were each lacking in 22 studies, with only one study providing both types of statements (Mwalabu et al., 2017) [54].

### Synthesis of the evidence

**Fig. 2:**
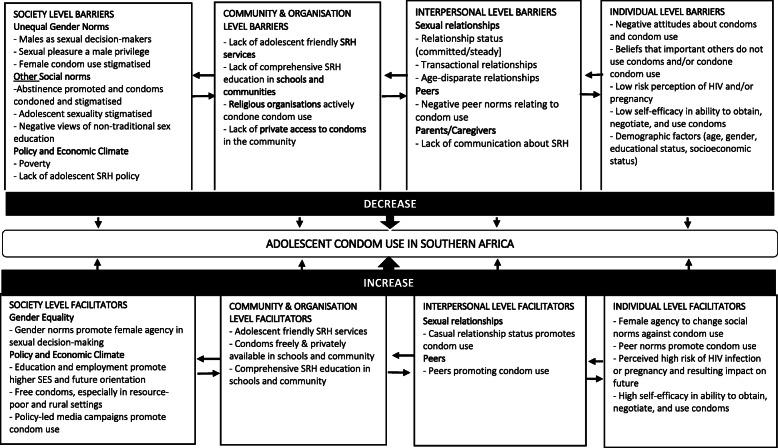
Multilevel model of barriers and facilitators of condom use among adolescents in Southern Africa

The findings provided evidence to support all 12 a priori themes in the original ‘best-fit’ model depicting the multi-level determinants of condom use among adolescents in Southern Africa (see Table 3). Further thematic analysis resulted in six inductive themes that revealed factors that can act as barriers or facilitators of condom use at the three levels in the model. A summary of the themes and sub-themes along with examples of supporting data are presented in Table 4 and a synthesis of the barriers and facilitators and their overlapping nature is presented in the conceptual model (Fig. 2).

**Table 3:** A Priori Themes – Individual, interpersonal and structural level determinants of condom use among adolescents in Southern Africa

**INDIVIDUAL LEVEL**		
**A Priori Themes/theoretical determinants**	**Key findings**	**Qualitative studies in the review citing influence of theoretical determinant**
**Sexual and Reproductive Health Knowledge** 10 studies indicated the relevance of adolescents’ knowledge about how to access and use condoms and the health risks and benefits of using condoms correctly and consistently.	*Key Finding: Inadequate SRH knowledge among adolescents* *Key Finding: Adolescents who had adequate SRH knowledge had engaged in SRH programmes at school or in the community*	- Three studies reported that sexual and reproductive health knowledge was evident among the adolescents in their studies [37, 40, 50]. Adolescents who had adequate SRH knowledge noted that they had engaged in SRH programmes at school or in the community [40, 50]. - Seven studies reported that inadequate or inaccurate SRH knowledge was common [38, 39, 47, 48, 50, 55, 57], mainly because participants had not received comprehensive SRH education.
**Attitudes and beliefs about condom use** 20 studies mentioned the relevance of attitudes (positive and negative) about condoms and condom use condoms.	*Key finding: Negative attitudes about condom use were reported as a key determinant. These included attitudes that condom use reduces sexual pleasure for men, is morally inferior to abstinence, promotes sexual promiscuity and a lack of trust in committed relationships.* *Key finding: A minority of studies reported positive attitudes towards condom use as a facilitator of condom use.*	- Twenty studies reported negative attitudes about condoms and condom use with the central attitudes being they are ineffective [38, 39, 41, 45, 52, 53, 57], cause disease [43, 51, 53, 56, 57], reduced sexual pleasure for men [39, 40, 42, 44, 45, 47, 53, 56, 57], are morally inferior to abstinence outside of marriage [41, 53, 59], and represent a lack of trust in committed and transactional relationships [37, 41, 42, 44, 46–49, 54, 59]. - Two studies did report positive attitudes to condoms (generally related to their value in preventing STIs and unintended pregnancy) [43, 49]. - Two studies reported perceptions that condom use indicated trust and respect in relationships [37, 59]
**Perceived barriers to condom use** 20 studies reported links between adolescent perceptions that people do not use condoms because of various psychosocial factors.	*Key Finding: Perceived barriers to condom use reported by adolescents included stigma, perceptions of reduced pleasure, not carrying condoms, beliefs about effectiveness, religious beliefs and the perceived impact of condom use on sexual relationships.*	- Three studies reported male adolescent beliefs that girls who carry or use condoms are ‘easy’, untrustworthy and likely suffering from a STI [38, 42, 46]. Six studies reported adolescent girls fear of embarrassment or judgment if they sought to obtain, carried or requested to use condoms [44, 46, 48, 51–53]. Three studies indicated that both males and females saw perceived stigma attached to adolescent sex as a barrier to obtaining condoms [51, 52, 59]. - Perceptions that condoms negatively impacted on pleasure or sexual satisfaction were noted in nine studies [39, 40, 42, 44, 45, 47, 53, 56, 57]. Although there were no reports from adolescent women regarding reduced pleasure, in one study young men claimed that their female partners complained that condoms bruise them [42]. - Two studies noted that a common barrier to condom use was that young people did not carry condoms with them and therefore did not have them readily available when they needed them [46, 57]. Both studies mentioned time and the space in which young people choose to have sex as relevant. - Seven studies reported that some adolescents did not use condoms because they believed they were ineffective in preventing HIV/STIs and pregnancy [38, 39, 41, 45, 52, 53, 57]. - Common negative beliefs were that condoms actually cause diseases such as cancer and other illnesses such as rashes, sores and stomach pains [43, 51, 53, 56, 57]. - Adolescents in three studies [41, 53, 59] noted that their religious beliefs acted as a barrier to condom use. - Six studies reported possible negative impacts on committed relationship dynamics as a barrier to condom use [37, 38, 40, 46, 47, 53] and five studies mentioned transactional relationships as barriers [41, 42, 44, 48, 59].
**Risk perception and beliefs about consequences of using/not using condoms** 12 studies reported beliefs about the consequences of using/not using condoms as determinants of condom use	*Key finding: Perceptions of risk of having to leave education and risk of contracting HIV from a casual partner were mentioned as a facilitator of condom use for some.* *Key finding: In this context, perceptions of risk of contracting condoms appeared to be moderated by perceptions that they were immune to catching HIV because of their choices or that they had become so used to HIV that they no longer feared it.* *Key finding: In age-disparate and transactional relationships risk perception appeared to be moderated by factors such as poverty and beliefs that condom use would result in a loss of the relationship.*	- Six studies mentioned finishing school and the importance of education as a belief that encouraged condom use among adolescent boys and girls [37, 40, 43, 49, 56, 57]. - Two studies mentioned that the fear of HIV when relationships were of a casual nature was a facilitator of condom use [46, 47] and one noted that younger adolescents seemed to fear the consequences of unprotected sex more strongly [49]. - One study [47] noted that young people appeared to think they were immune to HIV/AIDS because their lifestyle was such that they would not have sex without a condom with anyone whom they deemed to have HIV. Similarly, one study [57] highlighted that the young men in their study had become so used to HIV that the fear of contracting the illness was as low to them as the fear of catching the flu. - Although several studies highlighted that girls were aware of the risks of having sex with older men, this seem to be overpowered by their belief that not using condoms would result in negative consequences for them [41, 42, 44, 48, 59].
**Beliefs about ability to obtain and use condoms** (Perceived Behavioural Control) 7 studies reported a person’s confidence or lack of confidence in their ability to a) obtain condoms; b) negotiate their use with their partner; and c) use them correctly and consistently every time they have sex.	*Key finding: Knowledge about where to obtain condoms, self-efficacy obtaining condoms, costs of condoms, self-efficacy in ability to use condoms correctly presented as common* *Key finding: Beliefs about ability to negotiate condom use presented as a challenge for females*	- Adolescents in four studies reported that they did not know where to obtain condoms and others reported that, although they did know where to obtain them, they did not feel confident doing so [38, 44, 51, 57]. - Two studies reported low beliefs in ability to use condoms, an issue that was linked to a lack of comprehensive SRH education [44, 57]. - One study reported female adolescents’ low-self-efficacy to negotiate condom use with their partners, particularly older men [48] and another reported that high self-efficacy in ability to communicate with partners about condom use was a facilitator [59]. - One study highlighted that for some, lack of behavioural control was blamed on puberty [58].
**Intentions to use condoms** 12 studies presented findings relating to a person’s stated intention to use condoms when they have sexual intercourse.	*Key Finding: There was evidence from two studies to suggest that female agency was related to high intentions to use condoms* *Key Finding: Several studies indicated negative intentions among adolescents in committed, age-disparate or transactional relationships*	- Reports of intentions to use condoms generally related to female affirmations that regardless of possible barriers, they intended to use condoms any time they had sex [45, 46], intentions to use condoms whenever they had a sex with a new partner or a part who had not been tested for STIs [44], or intentions to use condoms in order to avoid future negative consequences for education, employment [37, 41, 42, 44, 46–49, 54, 59]. - Nine studies that indicated that adolescents (particularly males and females in relationships with older men) did not intend to use condoms, especially in committed or transactional relationships [37, 41, 42, 44, 46–49, 54, 59].
**Past Behaviour** 1 study presented findings relating to person’s past as a determinant of condom use	*Key finding: One study reported that past condom use behaviour could be a barrier or facilitator of condom use*	One study [57] noted that intentions to use condoms is associated with past condom use “Once you start having sex without a condom, you cannot change to using a condom. Sometimes someone will try to use a condom and not use a condom to compare the difference. So then in that moment, they forget about HIV and pregnancy because the temptation is so high to not use a condom.”
**Sociodemographic determinants of condom use** 10 studies reported sociodemographic factors as determinants of condom use	*Key finding: Being male presented as a determinant of condom use, with negative impacts more pronounced for older adolescents* *Key finding: Middle class males in education more likely to use condoms* *Key finding: Poverty as a determinant of condom use for young women (especially those in transactional relationships with older men)* *Key finding: Marriage a determinant of condom use*	- In general studies reported that male adolescents were less likely to use or want to use condoms than females [40, 49, 52, 57], although two studies reported that this was more common among older adolescents and older men, with younger boys and girls reported to be more likely to use condoms. - One study reported that young women assumed that it was less risky to have unprotected sex with younger than older men [42]. - Two studies noted that middle-class male adolescents and older adolescents still in education were more likely to express positive attitudes towards condom use [43]. -Several other studies noted that poverty was a barrier to condom use if free condoms were not provided [46] or in instances when young women agreed to sex with men in return for material goods [48, 49, 54, 59]. - One study mentioned that secondary school boys availed of free condoms because they feared getting their partner pregnant and had no money for an abortion [49]. - One study mentioned that unmarried adolescent women were more likely to mention condom use than married women [60].
**INTERPERSONAL LEVEL**		
**A Priori Themes/theoretical determinants**	**Key findings**	**Qualitative studies in the review citing influence of theoretical determinant**
**Interpersonal determinants of condom use** 14 studies described the barriers and facilitators of condom use at the interpersonal level. These related to relationship dynamics with sexual partners, peers and parents.	**Sexual Partners:** *Key Finding: Condom-use in casual relationships more widely accepted than condom-use in monogamous, transactional and age-disparate relationships* *Key Finding: There were some indications of a shift in sexual relationship dynamics*	- Six studies reported that for those in monogamous relationships, not using condoms appeared to represent trust, faithfulness and respect [37, 38, 40, 46, 47, 53]. - Two studies reported that those who requested condoms were assumed to be ‘sick’ or untrustworthy, especially women [42, 48] - Five studies noted that condom use was less acceptable in age-disparate and transactional relationships [41, 42, 44, 48, 59]. - One study reported that condom use was seen as a sign of respect for some [37]. - One study noted that early fatherhood acted a facilitator of condom use among some young men [40] and two others reported agency on the part of young women [45, 46].
	**Peers** *Key finding: Peer relationships can exert positive or negative influence on condom use*	- Two studies noted that negative peer norms relating to condom use acted as a barrier, particularly for young men [46, 47]. - One study reported peers acting as facilitators of condom use by sharing their condoms with friends [51].
	**Parents/Primary Caregivers** *Key finding: A lack of communication and guidance from parents/primary caregivers in relation to SRH was indicated as a possible barrier to condom use.*	The studies indicated a lack of communication between parents and adolescents about SRH, parental discomfort discussing sexual matters and adolescent perceptions that parents would disapprove of condom use [40, 46, 51, 57].
**STRUCTURAL LEVEL**		
**A Priori Themes/theoretical determinants**	**Key findings**	**Qualitative studies in the review citing influence of theoretical determinant**
**Organisational determinants of condom use** 18 studies described the determinants of condom use at the organisational or institutional level. These included SRH providers or clinics; religious organisations, schools and other organisations in the community including private enterprises such as guesthouses, bars and pharmacies	**Sexual and Reproductive Health providers or clinics** *Key finding: Some adolescents, particularly young women, report negative experiences with professionals and a lack of provision of easily accessible, privately available condoms at SRH clinics* *Key finding: Some SRH professionals report not wanting to distribute condoms because it might encourage ‘promiscuity’* *Key finding: Positive experiences reported with youth-friendly services*	- Three studies reported that staff did not distribute free condoms to adolescents because they did not want to encourage sexual activity [38, 54, 55] and three other studies reported negative experiences at clinics, which included being shouted at and judged by healthcare staff, particularly by young women [46, 51, 52]. - Two studies reported the facilitative effect of positive attitudes about condom use from health professionals [50, 51].
	**Religious organisations and their representatives** *Key finding: Acceptance of religious norms by adolescents and other community members can act as a barrier to condom use among adolescents*	Six studies reported that religious leaders encouraged abstinence and monogamy and condoned or discouraged condom use [38, 41, 43, 45, 53, 54]. - One study reported that an Archbishop had alleged that condoms had been infected by Western countries in order to ‘finish the African people’ [43]. - One study indicated that health professionals reported a conflict between promoting sexual wellbeing and conforming to religious norms [54] while another found that acceptance of religious norms had influenced the provision of SRH [38].
	**Schools** *Key finding: Absence of or inadequate SRH education in schools was reported as a barrier to condoms use among adolescents* *Key finding: Some studies note that inaccurate information to young people* *Key finding: Learning about SRH from peers or initiation ceremonies was common* *Key finding: Provision of condoms and SRH programmes in schools was noted as a facilitator*	Six studies mentioned the absence of sex education in schools as an organisational determinant of condom use, which often resulted in inaccurate knowledge and harmful sexual practices [39, 46, 47, 52, 55, 57]. - Two studies noted that information provided by existing SRH programmes was inaccurate, for example providing young people with false statistics relating to the efficacy of condoms [41, 59]. - Discomfort among teachers was noted by one study [57] as a possible reason for a lack of provision and a lack of SRH resources for teachers was noted in another study [52]. - Adolescents reported learning about SRH primarily from peers and initiation ceremonies [39, 55]. - One study noted the provision of condoms in schools as a facilitator of condom use [46] and another mentioned the provision of SRH programmes in schools as a facilitator [40].
	**Other community Organisations** *Key findings: The availability of condoms in organisations in the community was reported as a facilitator of condom use*	- Five studies found that organisations within communities acted as facilitators of condom use with bars [38, 52], guesthouses [58], and shops [44, 46, 52] often mentioned as places where young people had access to condoms.
**Society and community level determinants of condom use** 17 studies described the barriers and facilitators of condom use relating to social norms at level of community and society. These included gender norms and social norms.	**Gender Norms** *Key finding: The stigmatisation of condom use among adolescents in general, and young women in particular, is a key negative determinant condom use* *Key finding: Unequal gendered norms relating to sexual decision-making and responsibility, favouring men, is a determinant of condom use* *Key finding: Some studies reported a shift in thinking and disregard for unequal gendered norms among adolescents which acted as a positive determinant of condom use*	- Thirteen studies mentioned the influence of gender norms as determinants of condom use [37, 38, 40, 42, 45–49, 51–53, 59], with most highlighting that unequal gender norms impacted negatively on young people’s condom use. - Ten studies found that condom use among adolescent women was highly stigmatised [38, 40, 42, 46–49, 51–53], and it was also evident that restrictive masculinities were a negative determinant of condom use among men [40, 46, 52, 53]. - Six studies noted that men played the central role in making sexual decisions [40, 46–48, 53, 59]. -Two studies reported female as a positive determinant of condom use [45, 46] and in two other studies young men reported that they viewed using condoms as a sign of respect for their partners [37, 59].
	**Other Social Norms** *Key finding: Some studies reported that social norms which favour traditional methods of SRH education (including initiation ceremonies) and family planning acted as negative determinants of condom use*	- One study noted that young people received a lot of information about sex from traditional ‘initiation ceremonies’, which often led to inaccurate knowledge [39]. - Six studies mentioned that there was a desire for communities to retain their traditional culture and methods of avoiding HIV and pregnancy (which generally involves avoiding sex outside of marriage) rather than embracing contemporary ‘Western’ methods [38, 43, 44, 53, 54, 59].
**Political and economic barriers and facilitators of condom use** 13 studies described the determinants of condom use at the political and economic levels.	**Political and Economic:** *Key finding: There were indications that provision of free condoms and that national mass media campaigns to promote condom use was an important determinant.* *Key finding: Lack of an adolescent SRH strategy was identified as a barrier for educators wishing to incorporate RSE into their curriculum.* *Key finding: The availability of free condoms was noted as important, particularly in resource poor and rural settings.*	- One study [52] identified media advertisements as important in promoting condom use but also found that the lack of an adolescent sexual and reproductive health strategy as a barrier for educators wishing to incorporate RSE into their curriculum. - One study [46] highlighted the facilitating effects of government provision of free condoms, particularly in resource poor settings. One study highlighted that condoms were not available in some rural villages [44]. - Five studies reported the negative impact of poverty on young women’s decisions to use condoms in age-disparate and transactional relationships [41, 42, 44, 48, 52, 59]. - Conversely, six studies reported that higher socioeconomic status and future orientation were positive determinants of condom use [37, 40, 43, 49, 56, 57].

**Table 4:** Synthesis themes and sub-themes

Themes and sub-themes	Studies presenting evidence for theme	Examples of evidence for theme
**1. Unequal Gender norms and Restrictive Masculinities:** *Pervasive unequal gender norms and restrictive masculinities are barriers to condom use* ***Sub-themes:*** - Women that use condoms are ‘up to no good’ - ‘Real’ men don’t wear condoms - Sexual pleasure and decision-making a privilege of men - Female agency to change gendered norms	15/23 studies [38–40, 42, 44–49, 51–53, 57, 59] presented evidence for this theme.	*Social norms encroach on the extent to which young women are prepared to carry condoms with them. Participants mentioned that gossip is a constant source of conflict in the township and that women carrying condoms risked being labelled a ‘bitch’ or promiscuous* [46]. *Some girls also showed agency in refusing unprotected sex in cases where partners did not use a condom. In such cases, boyfriends would be requested to buy one; otherwise sex would not take place* [45].
**2. Other social norms:** *Social norms reflecting negative perceptions of non-traditional sexual and reproductive health education, non-traditional family planning, and adolescent sexuality* are *barriers to condom use* ***Sub-themes:*** -Traditional methods of family planning and education are best - Adolescent sexuality is taboo - Social norms are possible moderators of risk perception	20/23 studies [37–39, 41–50, 52–55, 57–59] presented evidence for this theme. Six of these studies mentioned that there was a desire for communities to retain their traditional methods of family planning [38, 43, 44, 53, 54, 59].	*“Malagasy tradition says that condoms are not good in some communities. I think Malagasy people think, ‘why should we change what we’ve been doing when it has been working? My ancestors weren’t sick with STIs so why should we have any problem?’”* [44] *“The fact of selling condoms encourages the widespread* [sic] *of sexual violence. It is as if people are conveying a message saying: ‘have sex and satisfy your sexual urge’. This can discourage men to hold their sexual urge until they get married, because they know that they cannot get diseases.”* [53] *“[Young people] know everything [about HIV] and may pass people in the street and even say ‘so and so is infected’. But the boys say, why are you afraid of HIV and not flu? They have become unafraid because it’s so normal.”* [57]
**3. Political and Economic Climate**: The political and economic climate are barriers and facilitators of condom use ***Sub-themes:*** -Policy-led promotion and resourcing of adolescent SRH and condom use - Poverty and socioeconomic status	14/23 studies [37, 40–44, 46, 48, 49, 52, 54, 56, 57, 59] presented evidence for this theme.	*There is still no policy or law that specifically caters for the reproductive health needs of adolescents in Zimbabwe. Consequently, this has made it difficult (…) for the Ministry of Education to develop comprehensive national adolescent reproductive health syllabi for the respective grades* [52]. *“Diseases will not end if they sell condoms to us. People here are very poor; if someone gets five rand they spend it on bread and candles, not condoms.”* [46] *“That’s what is happening with these schoolgirls we have. They’re financially dependent on these taxi drivers and all, because they are coming from poor backgrounds. There is no money at home. They need to buy cell phones and all these* [sic] *stuff. So in order to get that, she tells herself — Let me fall in love with the taxi driver, he’s going to provide all that for me. And if the taxi driver says — no condom.”* [41] *“[…] Such statements were common in middle class youth’s rationalisations of condom use and safe sex. Hence, the planning of sexual and reproductive behaviour was part of a broader view of life and future where the risk of HIV infection and pregnancy are lumped together as threats on the way towards fulling ones dreams and careers.”* [43]
**4. Community-based resources and influences:** Community-based organisations, facilities and spaces are barriers and facilitators of adolescent condom use ***Sub-themes:*** -Accessible adolescent SRH services - Sex education in schools and communities - Religious Influences - Access to condoms and spaces for sex in communities	18/23 studies [38–41, 43–47, 50–55, 57–59] presented evidence for this theme.	*“If you take any contraception at the clinic, you should know that before sunset everyone (including your parents and church members) in the community will be aware that you have taken some contraceptives at the clinic. This will be definitely the hot selling news of the day. The news will spread like a wild fire accompanied by a mighty wind.”* [52] *“We promote the natural method for doing family planning... this method is the one proposed by the Catholic Church because the artificial methods do not conform with the will of God.”* [38]
***5.*** **Interpersonal Influences:** *Interpersonal relationship dynamics are barriers and facilitators of condom use* ***Sub-themes***: - Trust and Transactions in Sexual Relationships - Peer Influences - Parent/Caregiver Communication	13/23 studies [37, 38, 40–42, 44, 46–48, 51, 53, 54, 59] presented evidence for this theme.	*“….he has been supporting me since I was 10 years old. I felt like paying back in kind (exchanging his kindness with sex); how about transmitting the virus to him? How could I suggest condom use? If he knew my [HIV] status, I felt like losing my SACCO (Savings and Credit Cooperative – a money lending agency in Malawi); I conceived…he married me.”* [54] *“If you’re unsure of your husband, you need to buy condoms.”* [59] *Many of the* [male] *participants stated that they had been accused of being stupid* [and jeered at by their friends] *after using condoms and had decided that they would not use them again* [46]. *Teenagers don’t want to be seen obtaining condoms because they don’t want their parents to know that they are sexually active (even though adolescent sexual activity is the norm)* [51].
**6. Adolescent attitudes about condoms:** *Negative views of condoms and condom use among adolescents as barriers to condom use* ***Sub-themes:*** -Male pleasure and performance -Condom myths	14/23 studies [38–45, 47, 51–53, 56, 57] presented evidence for this theme.	*“Young men socially construct their ideal of true love in unprotected or ‘flesh-to-flesh’ sex. Nowadays’ youth often say: ‘you cannot eat a candy in a wrapper’”* [53]. *“I realized that even if you use thousand condoms at once, the way that they are built can cause cancer. You can get sicknesses or pregnancy though you wear them, they can prevent absolutely nothing.”* [53]

#### Barriers and facilitators of condom use among adolescents in southern Africa

##### Theme 1: unequal gender norms and restrictive masculinities

Pervasive unequal gender norms and restrictive masculinities were identified barriers to condom use among adolescents in Southern Africa in the reviewed studies, with some emerging evidence of shifting norms. Examples of these barriers include stigmatisation of adolescent women who use condoms and unequal gendered norms relating to sexual decision-making, responsibility and pleasure that favoured young men. While it was reported that condom use among adolescent women was highly stigmatised and neither acceptable nor expected of women of ‘good’ moral character [38, 40, 42, 46–49, 51–53], it was also evident that there was widespread concurrence with the notion that ‘real’ men do not use condoms [40, 46, 52, 53].

Reflecting these unequal gender norms, adolescent boys and young men in several studies reported a belief that girls who carry or use condoms are ‘easy’, untrustworthy and likely suffering from a sexually transmitted infection (STI) [38, 42, 46]. Similarly, studies reported adolescent girls’ fear of embarrassment or judgment if they sought to obtain condoms, or if they carried or requested to use condoms [44, 46, 48, 51–53]. For example, one participant in a Zimbabwean study indicated that if she requested for her male partner to use a condom *“he* [would] *think that you were up to no good, you were having sex with many people previously.”* [48].

Several studies noted that adolescent boys and young men played the central role in sexual decision-making in the study countries [46–48, 53, 59]. When adolescent girls and young women took ‘responsibility’ for condom use, they also take took ‘blame’ for engaging in taboo behaviours. The author of one Mozambican study notes:

*‘Girls have to decide for themselves if they will take the responsibility for carrying condoms. In doing so, they can avoid risk-behaviour and lessen the male’s sense of duty. At the same time, girls have to face embarrassment when the initiative of carrying condoms comes from the female partner.’* [40]

Unequal gender norms as a barrier to condom use were also evident in widespread reporting of sexual pleasure as a privilege of men [39, 40, 42, 44, 45, 47, 56, 57, 61]. A contradiction in this regard is highlighted in a Mozambican study: although young men believed condoms reduced pleasure, they would still use condoms with sex workers because of the perceived heightened risk of HIV. The author notes ‘*Condoms only appear to interrupt pleasure in close and steady relationships, and not with people considered more likely to be HIV infected’* [47].

Several studies suggested a shift in thinking that might facilitate condom use, with young men in one study reporting that they viewed using condoms as a sign of respect for their partners [37], and others reporting shifts in thinking in relation to gendered norms, specifically female agency as a facilitator of carrying and using condoms [45, 46, 59]. There were no clear indications or explanations offered for why or how these shifts were occurring.

##### Theme 2: other social norms

The included studies also highlighted the existence of other social norms that acted as barriers to condom use. These included 1) the stigmatisation of adolescent sexuality and non-traditional family planning and SRH education and 2) social norms that appeared to act as moderators of risk perception.

Social norms that favoured traditional methods of family planning or SRH education that was not evidence-based were reported as barriers to condom use. Several studies mentioned norms promoting traditional practices and methods of avoiding HIV and pregnancy (which generally involved avoiding sex outside of marriage) rather than embracing contemporary methods, which were often viewed more negatively [38, 43, 44, 53, 54, 59].

Social norms reflecting negative perceptions of adolescent sexuality were suggested in several studies; stigma attached to adolescent sexual activity was reported as a barrier to both male and female adolescents’ ability to obtain condoms [51, 52, 59]. The pervasive nature of these norms was evident in organisations that prevented young people from accessing free condoms or judgement-free SRH services [38, 46, 50–52, 54, 55], and inadequate provision of accurate SRH education in schools or communities [39, 46, 47, 52, 55, 57]. A respondent from one Zimbabwean study noted, *“How can you take a condom from the collection points at the clinics when all patients (some friends, relatives, and teachers, religious and traditional leaders) in all age groups will be staring at you?”* [52].

There was also evidence suggesting that social norms may act as a moderator of risk perception relating to HIV. In one Tanzanian study [57], it appeared that some young people had normalised HIV as something they should not fear because it was so prevalent in their communities. A participant notes: *“[Young people] know everything [about HIV] and may pass people in the street and even say ‘so and so is infected’. But the boys say, why are you afraid of HIV and not flu? They have become unafraid because it’s so normal.”*

Relatedly, and more common, were indications that adolescents associated HIV with those of ‘bad’ moral character and were therefore themselves immune to it because their ‘steady’ sexual partners were of ‘good’ moral character, like themselves [37, 41, 42, 44, 46–49, 54, 57, 59]. This was illustrated by a participant in one South African study: *“It’s if you have two girlfriends, your steady and your secret lover. You can never use a condom with your steady but you can use one with your secret lover because you don’t know if she has a disease.”* [46]*.*

##### Theme 3: political and economic climate

Political buy-in to the promotion of adolescent SRH was implicated as a potential facilitator of condom use among adolescents in some studies. These included policy-led provision of free condoms and SRH services in resource-poor and rural settings in South Africa and Madagascar [44, 46]. One Zimbabwean study author [52] noted that a lack of adolescent SRH legislation or national policy was a barrier for educators wishing to incorporate RSE into school curricula. Conversely, a government-backed national media campaign promoting condom use in Zimbabwe was reported as a facilitator [52].

Poverty and socioeconomic status were also noted as potential moderators of adolescent knowledge, attitudes, behavioural control and perceptions of risk. A common finding was the impact of poverty that led young women having to forego using condoms in age-disparate and transactional relationships [41, 42, 44, 48, 59]. Conversely, higher socioeconomic status and associated future orientation emerged as facilitators of condom use, and these factors appeared to mediate adolescents’ perception of risk. Several studies indicated that not being able to finish school due to pregnancy or HIV was the primary consideration among adolescents, with younger adolescents and those of higher socio-economic status reporting more strongly that they feared the consequences of unprotected sex [37, 40, 43, 49, 56, 57]. One Mozambican study author indicated ‘*Many boys expressed the idea that it was important to use condoms because ‘life must be protected when you have something to live for, something you want to do in your life’.* [43].

##### Theme 4: community-based resources and influences

Service- and community-level barriers and facilitators were reported across the 23 studies and included SRH services, religious organisations, schools, and the physical features of communities that influenced both access to condoms and the spaces in which young people have sex.

#### Accessible adolescent SRH services

A lack of adolescent-friendly, community-based SRH services frequently was presented as a barrier to condom use [38, 46, 50–52, 54, 55]. Some adolescents reported that they did not know where to obtain condoms in their communities, and others reported that although they had this knowledge they did not feel confident doing so because they feared judgment from clinic staff [38, 44, 51, 57]. Reflecting the influence of social norms, including gender norms, several studies reported that health professionals did not distribute condoms freely, privately or without judgment because they did not want to encourage sexual activity in adolescents [38, 46, 51, 54, 55]. Negative experiences at clinics were reported particularly by young women [46, 51, 55]. Conversely, one Tanzanian study reported the facilitative effect of positive attitudes about condom use from health professionals and emerging changes in social norms [50], and another study in Botswana reported positive experiences at a clinic where ‘*boxes of condoms were kept at* [an open] *window and no questions were asked [of adolescents who took them]*’ [51].

#### Sex education in schools and communities

Several studies mentioned the absence of sex education in schools and communities as a barrier to condom use [39, 46, 47, 52, 55, 57]. Inadequate or inaccurate SRH knowledge was common [38, 39, 47, 48, 50, 55, 57], mainly because participants had not received comprehensive SRH education [40, 50].

Discomfort among teachers presenting SRH material was noted by one Tanzanian study [57] as a possible reason for a lack of sex education provision, and a lack of SRH resources for teachers was noted by a Zimbabwean study [52]. Other studies indicated that information provided by existing programmes was inaccurate, such as giving young people false statistics relating to the efficacy of condoms [41, 59]. Adolescents in Zambia and Malawi reported learning about SRH primarily from peer discussion [39, 55]. Some studies noted the provision of condoms in South Africa [46] and SRH programmes [40] in schools in Mozambique as facilitators of condom use.

#### Religious influences

Religious organisations and their representatives were mentioned by several studies as barriers to condom use [38, 41, 43, 45, 53, 54]. The studies reported that religious leaders encouraged abstinence and monogamy, discouraging condom use because of beliefs that they encourage sexual promiscuity and/or because the use of birth control is not in line with their faith.

In one Malawian study, health professionals reported experiencing conflict between promoting sexual wellbeing and conforming to religious norms [54],while a Congolese study noted that acceptance of religious norms had negatively influenced the provision of SRH [38], although the quality of this study was impacted by a lack of clarity regarding the flow of conclusions from analysis: *“Nor were adolescents given full information, even about the menstrual cycle, on the grounds that it might encourage sexual liberties”* [38].

#### Access to condoms and spaces for sex in the community

Two studies noted that a barrier to condom use was that young people did not carry condoms with them and therefore did not have them readily available when needed [46, 57]. Relatedly, a Tanzanian study [58] reported that time and space are often barriers to young people so they choose to have sex in alleyways, cemeteries, toilets in bars, or unfinished houses. The authors suggest that due to fear of being interrupted young people are in a hurry and consider condom use a waste of time. *“*[They] *have sex in an unfinished house (…) they are not using condoms because they are in a hurry and they do not have time.”* [58] Similarly, in a South African study [46] adolescents reported having sex at home when their parents were out and not wanting to waste time by using a condom. In urban settings, organisations within communities including bars [38, 52], guesthouses [58], and shops [44, 46, 52] where young people had access to condoms were often mentioned as facilitators of condom use.

##### Theme 5: interpersonal influences

Across studies, interpersonal networks including sexual partners, peer influences and parent/caregiver communication presented as barriers and facilitators of condom use.

#### Trust and transaction in sexual relationships

Heavily influenced by unequal gendered norms, condom use within a sexual relationship was seen as dependent on the status of the relationship, with condom use in casual relationships more widely accepted than in committed relationships. For people in committed relationships, not using condoms appeared to be linked to trust, faithfulness and respect for one’s partner [37, 38, 40, 46, 47, 52, 53] and those who requested condoms were assumed to be ‘sick’ or untrustworthy, especially women [42, 48]. One study suggested an alternative perception, reflecting emerging changes in gender norms, of how condom use in committed relationships could be seen as a sign of faithfulness when a male partner uses a condom because his female partner does not want to get pregnant and therefore drop out of school [37]. Some studies indicated that age-disparate and transactional relationships acted as barriers to condom use, with older men reportedly less willing to use condoms and young women powerless to demand they do so, especially when the older man provides them with material support [41, 42, 44, 48, 59].

#### Peer influences

Some studies mentioned the importance of peer influences on condom use, noting that negative peer norms relating to condom use acted as a barrier, particularly for young men [46, 47]. It also appeared that peer norms also negatively affected condom use among females. One author of a South African study indicates: ‘*Young women argued that for a steady partner to insist on condom use is seen as indicating a lack of respect and trust that could destroy one’s reputation within the peer group. If a boy wants to use a condom she will say it is because he disrespects her, because he wants to use ‘a plastic’.’* [46] One Botswanan study reported male peers acting as facilitators of condom use by sharing their unused condoms [51].

#### Parent/caregiver communication

A lack of communication and guidance from parents and primary caregivers about SRH was indicated as a possible barrier to condom use by four studies [40, 46, 51, 57], particularly because of parental discomfort discussing sexual matters and adolescent perceptions that parents would disapprove of condom use [51].

##### Theme 6: adolescent attitudes about condoms

Attitudes that condoms negatively affecting pleasure or sexual satisfaction were noted as barriers to their use [39, 40, 42, 44, 45, 47, 53, 56, 57], with adolescent men claiming that condoms were too tight, painful, reduced their ability to maintain an erection, delayed orgasm and reduced sensation. Some said that using condoms reduced the ‘thrill’ of sex [53, 56, 57]. Reflecting the possible unequal impact of gender norms in relation to attitudes about condoms, there were no reports from adolescent women regarding reduced pleasure.

It also appeared that attitudes about condoms were strongly influenced by inaccurate knowledge. Studies reported that adolescents did not use condoms because they believed they were ineffective against STIs/HIV or pregnancy because they had ‘little holes’ in them allowing sperm (and HIV infection) to pass through [38, 39, 41, 45, 52, 53, 57]. Some adolescents reported that free condoms obtained at clinics were particularly defective [51, 52]. Related to this, some studies reported common attitudes that condoms cause disease illness such as cancer, rashes, sores and stomach pains [43, 51, 53, 56, 57].

## Discussion

This review aimed to synthesise qualitative evidence relating to the individual-, interpersonal- and structural-level barriers and facilitators of condom use among adolescents in Southern Africa. We identified and mapped relevant qualitative literature onto an a priori framework incorporating three theoretical models – the social ecological model [31, 32]; an integrated model of condom use among young people in sub-Saharan Africa [20]; and the *If Were Jack* theory of change [28–30] – and then used inductive thematic analysis to identify the key barriers and facilitators of condom use which we incorporated into a new conceptual model (Fig. 2).

The findings provided evidence to support all 12 a priori themes in the original ‘best-fit’ model depicting the multi-level determinants of condom use among adolescents in Southern Africa. The combined three-model framework was able to capture more determinants than any one of the models could accurately achieve on its own, thus suggesting that a multilevel conceptual model as outlined in Fig. 2 may be more appropriate.

In line with previous reviews [14, 62, 63], the findings indicate that young people in Southern Africa face a range of barriers to condom use operating at all levels of their social ecological milieu. Results also highlight complex interactions among six key themes and illustrate that both positive and negative influences are reinforced at different levels. In line with the UN Sustainable Development Goals and their focus on multilevel determinants of health, this review offers understanding and insights into how common themes such as gender inequality and poverty exert their influence on condom use across social ecological levels.

The findings of our review are consistent with other studies [14, 18, 62–64] whose authors have identified a need to move beyond individual-level behaviour change frameworks and programmes by incorporating broader understandings of the structural-level barriers and facilitators of condom use among young people in Southern Africa. We advance previous findings by offering a conceptual framework of the key barriers and facilitators at society, community, interpersonal and individual levels that might act as a guide for decision-makers, programme developers and researchers wishing to optimise intervention impact. We are also optimistic about the fact that we have uncovered positive findings relating to shifts in unequal gendered norms, with key messages relating to female agency in sexual decision-making emerging as key.

The findings suggest that valuable and limited resources should be best used in coordinated effort to increase condom use by implementing multilevel interventions at all levels of the social ecological system. This could be informed by emerging broad-based guidance for improving uptake and access to contraception [64]. Programming efforts should, at the very least, ensure efforts are made to start addressing the widespread influence of gender inequalities and restrictive gendered and social norms that appear to operate at all levels of the social ecological system. While this approach is not a panacea—especially in challenging political and economic climates which are characterised, at times, by a lack of policy or policy implementation relating to gender equality —it might help shift attitudes and initiate change slowly across the community, organisational, interpersonal and individual levels [21].

The findings of this study indicate the need for easily accessible and free condoms, especially in resource-poor and rural settings. Importantly, they equally emphasise the need for evidence-based adolescent SRH strategies that provide health professionals and educators training and resources needed to provide youth-friendly SRH services and education. The findings also highlight the influence of social norms reflecting negative perceptions of adolescent sexuality, and non-traditional sex education and methods of avoiding HIV, STIs and unintended pregnancy. The strength of these prevailing norms suggest that programme developers need to involve local stakeholders in co-design and co-production processes. Interventions that can engage wider community members where these negative norms are played out are also critical. Community mobilisation interventions have shown positive impacts in relation to other SRH interventions, particularly those that involve comprehensive SRH education and positive role modelling by community members such as community elders, religious leaders, healthcare professionals and educators [65–67]. Coupled with this approach is the need for individual-level interventions targeting health professionals, educators and community members that promote youth-friendly, evidence-based comprehensive SRH education and practice [68]. Decision-makers should also be lobbied to support such interventions with policy-led adolescent SRH strategies and to provide resources for their implementation with youth [69–71].

Centrally, comprehensive sex education delivered to adolescents in school and community settings should include gender transformative components; directly challenge stigma and myths surrounding condoms and condom use; and harness the facilitating effects of promoting female agency in sexual decision-making [21, 72]. These curricula should prioritise key issues such as male roles and responsibilities in relation to avoiding HIV and unintended pregnancy; sexual pleasure (particularly gender disparities in relation to expectations of pleasure and the contradictions relating to pleasure in committed versus casual relationships); and negotiating condom use (particularly in age-disparate and transactional relationships). Programmes that promote the roles and responsibilities of sexual partners, peers and parents/caregivers as key interpersonal influences will also be necessary [70].

### Strengths and limitations of the review

Core strengths of this review include the systematic process by which we integrated and analysed rich contextual data from a broad range of qualitative studies conducted with adolescents in Southern Africa, and used them to develop an integrated conceptual framework that might be of value for intervention developers and decision-makers.

Limitations include the possibility that we did not identify potentially relevant articles despite our comprehensive search. In particular, due to resource constraints, we excluded studies not reported in English as well as grey literature. We may therefore have missed studies that were relevant to the review. In particular, over the past two decades, ministries of education and health in many Southern African countries have worked to implement comprehensive relationships and sexuality education policies and programmes in schools and communities that have not been captured by this review. Further, while we report findings from 11 countries in Southern Africa, five other countries in the SADC region included in our search (Angola, Comoros, Lesotho, Mauritius and Seychelles) were not represented in the review. These omissions mean that the findings may not be entirely representative of the experiences of young people across the region. Additionally, due to uneven reporting of sample composition and age range, it was difficult to determine what findings might apply to sub-populations of adolescents. Further, because some factors were only reported on by studies in some of the included countries, we cannot assume that the findings are generalizable to all countries or populations in Southern Africa.

Finally, despite promising findings regarding quality in other domains, the lack of reflexivity related to theoretical and/or cultural positionality of the researcher is important to address in qualitative research, especially regarding sensitive content such as condom use and sexual practices. While bias can be mitigated in straightforward ways in quantitative research, researcher positionality is often not clearly stated, which can negatively influence the rigour and accuracy of the findings. Indeed, while we ourselves are an international, multiracial, mixed gender, multidisciplinary research team, we recognise that our interpretation of the findings represents but one among other possible interpretations. The data synthesis and drafting of this paper was led by the first author, a white, Irish, female, PhD, who positions herself as a social psychologist heavily influenced by interpretivism and realism. While we are confident that reflexive journaling as part of the analysis process, as well as team discussions drawing out various interpretations of the findings, have increased rigour, unconscious bias may have been inadvertently introduced.

## Conclusion

Our findings suggest that programmes should address the wider structural influences through both individual- and structural-level interventions, in addition to targeting individual-level socio-cognitive factors. It is especially important to understand the key barriers and facilitators of condom use that act as moderators on the pathway towards consistent condom use among adolescents. In Southern Africa, as in many other regions of the world, these barriers include unequal gender norms and restrictive masculinities that stigmatise adolescent girls; social norms that restrict positive sexualities among adolescents and perpetuate negative perceptions of condoms and condom use; and the pervasive impact of poverty on access to condoms, SRH education and the power to negotiate condom use freely. SRH programming targeting barriers and facilitators of condom use at multiple levels is urgently needed in Southern Africa. Such programmes should pay particular attention to the key gaps in knowledge and evidence-based interventions highlighted by this review, for example: promotion of female agency in SRH decision-making; increasing positive parent-child communication about SRH; increasing accessible youth-friendly SRH services; and encouraging the widespread adoption and implementation of strategies to improve SRH.

## Supplementary Information

**Additional file 1.** Search Terms.

**Additional file 2.** Coding Framework.

## Data Availability

The review protocol is available in Prospero. The datasets used during the current study have not been stored in a public database. They are available from the corresponding author on reasonable request.

## Abbreviation

SRH: Sexual and Reproductive Health
