# Supplementary material for: Adolescent condom use in Southern Africa: narrative systematic review and conceptual model of multilevel barriers and facilitators
Source: BMC Public Health. 2021 Jun 26;21:1228. doi: 10.1186/s12889-021-11306-6 (PMC8234649; doi:10.1186/s12889-021-11306-6)
Supplement: Supplementary file 1 — Additional file 1. Search Terms. [file 12889_2021_11306_MOESM1_ESM.docx]

## Additional File 1: Search Terms

| 1 | (Adolescen* OR Juvenile* OR Student OR Pupil OR Learner OR Teen* OR Young adult OR Young people OR Youth OR Young wom* OR Young men OR Young man OR Pubescent).ab,ti,sh. |
| --- | --- |
| 2 | (Determinan* OR factor* OR reason OR predictor OR construc* OR cause OR associate* OR correlat* OR intention OR intend OR barrier OR social cognitive model OR health belief model OR theoryADJ2planned behavio*). ab,ti,sh. |
| 3 | Individual OR intrapersonal OR interpersonal OR Personal OR gender OR health status  OR Family OR Peer OR friend* OR house OR household OR Community OR school OR social OR relational OR civil OR culture OR environment* OR religou* OR society OR organizational |
| 4 | (Condom* OR contracept* OR Safe sex OR Unsafe sex OR Protected sex OR Responsible sex OR Unprotected sex OR Safe intercourse OR Unsafe intercourse OR Protected intercourse OR Unprotected intercourse OR protection).ab,ti,sh. |
| 5 | (Southern Africa OR Angola OR Botswana OR Comoros OR Congo OR DRC OR Eswatini OR Lesotho OR Madagascar OR Malawi OR Mauritius OR Mozambique OR Namibia OR Seychelles OR SouthADJAfrica OR Tanzania OR Swaziland OR Zambia OR Zimbabwe).ab,ti,sh. |
| 6 | (SouthADJAfrica* OR South Africa* OR Lesotho).ab,ti,sh. |
| 1^st^ option | 1 AND 2 AND (3 OR 4) AND 5 |
| 2^nd^ option | 1 AND 2 AND (3 OR 4) AND 7 |

## 
