# Supplementary material for: Adolescent condom use in Southern Africa: narrative systematic review and conceptual model of multilevel barriers and facilitators
Source: BMC Public Health. 2021 Jun 26;21:1228. doi: 10.1186/s12889-021-11306-6 (PMC8234649; doi:10.1186/s12889-021-11306-6)
Supplement: Supplementary file 2 — Additional file 2. Coding Framework. [file 12889_2021_11306_MOESM2_ESM.docx]

## Additional File: 2 Coding Framework

| **Concepts derived for coding** | **Definitions** |
| --- | --- |
| **Individual level** |  |
| Sexual and Reproductive Health Knowledge | Accuracy of a person’s knowledge about how to access and use condoms and the health risks and benefits of using condoms correctly and consistently e.g. I know how to use a condom, I know that condoms prevent HIV and pregnancy. |
| Attitudes and beliefs about condom use | A person’s attitudes and beliefs (positive and negative) about condom use e.g. ‘condoms are good because they prevent sexually transmitted infections’ or ‘condoms are bad because they reduce pleasure’. |
| Perceived barriers to condom use | A person’s perception that people do not use condoms because of… false beliefs/myths (e.g. condoms spread disease); stigma (e.g. only prostitutes carry condoms); beliefs about pleasure (e.g. condoms reduce pleasure); beliefs about effectiveness (e.g. condoms don’t work); religious beliefs (e.g. Catholics don’t use condoms); lack of consent (e.g. in cases of sexual violence and coersion, under influence of alcohol or drugs); relationship dynamics (e.g. condoms are not necessary in committed relationships). |
| Risk perception and beliefs about consequences of using/not using condoms | A person’s belief that not using condoms will result in negative consequences for their current or future lives (e.g. disease, unintended pregnancy, loss of educational or occupational opportunities) or that using condoms will help avoid negative consequences. |
| Beliefs about ability to obtain and use condoms | A person’s confidence or lack of confidence in their ability to a) obtain condoms; b) negotiate their use with their partner; and c) use them correctly and consistently every time they have sex. |
| Intentions to use condoms | A person’s stated intention to use condoms when they have sexual intercourse.  A person’s intention to ensure that if they are not using condoms, they and their partner has been tested for STIs and they are using another method of contraception. |
| Past Behaviour | A person’s past behaviours (e.g. sexual activity, substance use, criminal/delinquent behaviours) as a perceived reason for why they do or do not use condoms e.g. it’s difficult to start using condoms if you’ve never used them before; people under the influence of alcohol or drugs are less likely to use condoms. |
| Sociodemographic influences | A person’s age, gender, ethnicity, location, relationship status (married/single; casual/committed), or socioeconomic status (income, education) as a perceived reason for why they do or do not use condoms e.g. younger boys more likely to use condoms; adolescents from poorer backgrounds less likely to use condoms; married women are less likely to use condoms; those from certain regions or rural areas less likely to use condoms. |
| **Interpersonal level** |  |
| Interpersonal determinants of condom use | Parent-child communication and supervision  Sexual relationships: status (casual v committed), age-disparate relationships, coercive relationships, transactional relationships  Peer influences/peer pressure |
| **Structural level** |  |
| Organisational determinants of condom use | *Schools*: Quality of education in general; Provision of RSE; Violence in schools  *Health services*: Youth friendly SRH services; availability of condoms; provision of free condoms; attitudes of health professionals  *Churches*: Actively encourage or discourage condom use  *Other*: Youth groups, NGOs provide SRH programmes to promote condom use |
| Social norms and values relating to condom use | *Gender norms:* Females should not carry or request to use condoms; Males make all decisions relating to sex; ‘Real’ men don’t use condoms; both males and females and roles and responsibilities in relation to SRH decision-making  *Social norms*: Sex outside of marriage is forbidden/frowned upon; we don’t use condoms in our tradition; we prefer our traditional ways of avoiding STIs and pregnancy  *Peer norms*: None/all of my peers use condoms  *Familial norms:* My parents encourage/discourage my using condoms |
| Political and economic determinants of condom use | Government led policies and resources to promote condom use  Community social and economic resources and programmes to promote condom use  Economic stability and livelihood opportunities, poverty, violence, infrastructure, modernisation  Stage of HIV/AIDS epidemic  Household assets and structure |
